# Uma Combinação Fatal: Indometacina e Dabigatrana

**DOI:** 10.36660/abc.20180159

**Published:** 2020-05-11

**Authors:** Adem Adar, Orhan Onalan, Fahri Cakan

**Affiliations:** 1 Karabuk University Faculty of Medicine Karabuk Turquia Karabuk University Faculty of Medicine – Cardiology, Karabuk – Turquia

**Keywords:** Indometacina/administração e dosagem, Dabigatrana/administração e dosagem, Cardomegalia, Derrame Pleural, Insuficiência Renal, Ecocardiografia

## Introdução

Embora raras, diversas complicações hemorrágicas podem ocorrer em pacientes sob uso de dabigatrana. O risco de hemorragia é particularmente alto em pacientes com insuficiência renal ou em uso concomitante de medicamentos nefrotóxicos.^1^ Relatamos um caso de derrame pleuropericárdico maciço desenvolvido após o início do tratamento com indometacina em paciente em uso de dabigatrana devido a trombose venosa profunda.

## Relato de caso

**Figura 1:**
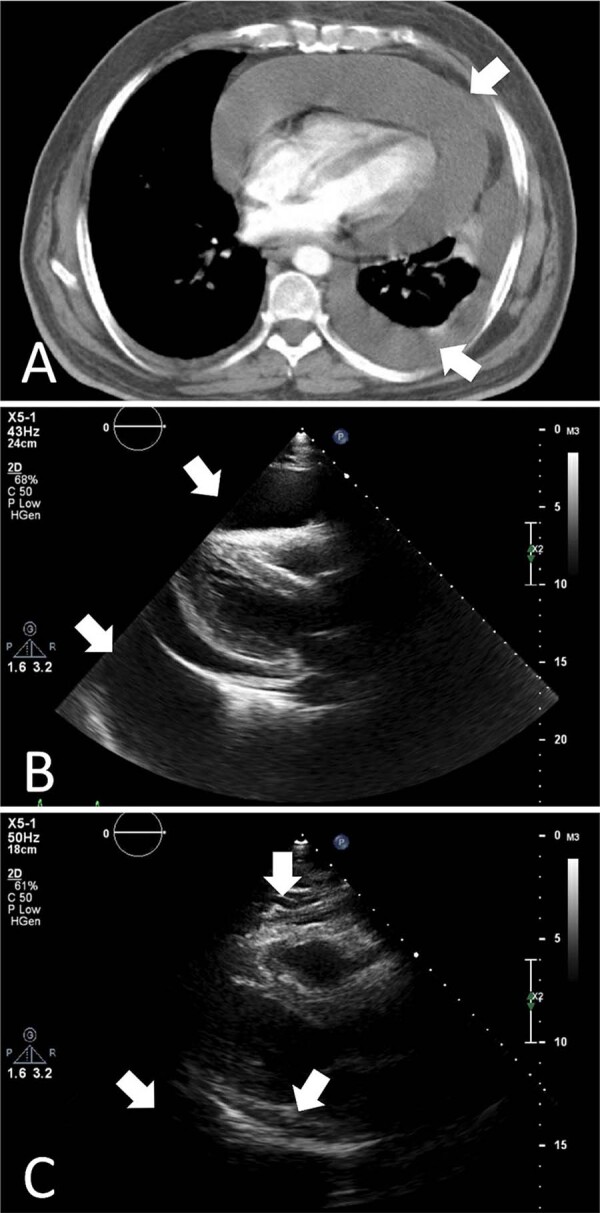
– A) Derrame pericárdico e pleural maciço na tomografia torácica. B) Derrame pericárdico ao redor do coração e derrame pleural. C) Derrame pericárdico completamente drenado.

**Figura 2:**
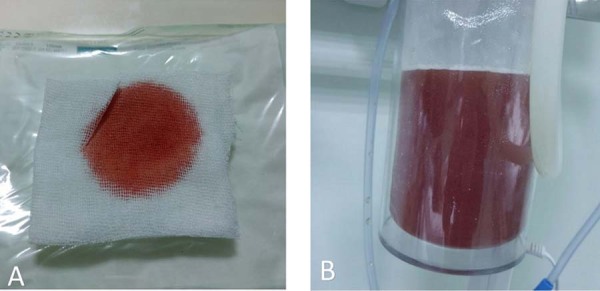
– Líquido pericárdico não coagulado vermelho-sangue.

Paciente do sexo masculino, 50 anos de idade, deu entrada no atendimento de emergência com dispneia progressiva. Apresentava frequência cardíaca de 120 batimentos/min, pressão arterial de 180/90 mmHg, frequência respiratória de 15 respirações/minuto, saturação de oxigênio de 95% (no ar ambiente) e temperatura de 36,8°C na internação. O paciente apresentava sedentarismo, obesidade (índice de massa corporal: 31 kg/m^2^), hipertensão não controlada (por 5 anos sem terapia médica) e trombose venosa profunda (em uso de dabigatrana 150 mg duas vezes ao dia por 50 dias). Vinte dias antes de sua internação, começou a receber indometacina (uma vez ao dia) devido à sua dor na perna. No exame físico, apresentava bulhas cardíacas e pulmonares diminuídas. A eletrocardiografia mostrou taquicardia sinusal. Radiografia de tórax mostrou cardiomegalia e derrame pleural bilateral (maiores no pulmão esquerdo). A tomografia computadorizada de tórax confirmou derrame pleural bilateral e revelou derrame pericárdico maciço ( Figura 1A ). Na admissão, os exames de sangue mostravam: glicose: 107 mg/dL, ureia: 63 mg/dL, creatinina: 1,99 mg/dL, AST: 69 U/L, ALT: 99 U/L, glóbulos brancos: 9,73 10^9^/L, hemoglobina: 9,6 mg/dL, proteína C-reativa: 0,9 mg/dL, tempo de tromboplastina parcial ativada (APTT): 91,4 segundos e razão normalizada internacional (RNI): 2.5. Como seu último nível de creatinina foi de 1,1 mg/dL, 20 dias antes (imediatamente antes do início do tratamento com indometacina), considerou-se insuficiência renal aguda. O paciente foi internado na Unidade de Terapia Intensiva, onde se realizou ecocardiografia detalhada. O ecocardiograma transtorácico mostrou função sistólica ventricular esquerdo normal (FE 65%), hipertrofia concêntrica ventricular esquerda (HCVE: 118 g/m^2^), derrame pericárdico e pleural maciço ( Figura 1B ). Não havia sinais de tamponamento cardíaco na primeira avaliação ecocardiográfica. No entanto, durante o acompanhamento, sua dispneia e taquicardia aumentaram gradualmente, e observou-se colapso diastólico ventricular direito na segunda ecocardiografia. Decidiu-se realizar pericardiocentese urgentemente. A fim de reduzir o risco de hemorragia, administrou-se idarucizumabe (total de 5 gramas divididos em duas infusões consecutivas de 2,5 gramas) antes da pericardiocentese. Duas horas após a administração de idarucizumabe, o valor do APTT caiu para 44 segundos. A pericardiocentese foi realizada com orientação ecocardiográfica. Aproximadamente 3 L de líquido pericárdico não coagulado vermelho-sangue foram drenados ( Figura 2 ). A análise do líquido pericárdico mostrou-se negativa para coloração de gram, citologia, PCR e microrganismos ( *Mycobacterium tuberculosis* ). Não houve derrame pericárdico na outra ecocardiografia realizada no dia seguinte após a pericardiocentese ( Figura 1C ). De acordo com os critérios de Light modificados, o derrame pericárdico apresentava características exsudativas. A toracocentese foi então realizada e 2 L de líquido pleural foram drenados. Os testes bioquímicos mostraram-se novamente consistentes com o líquido exsudativo. Os marcadores inflamatórios, reumatológicos, infecciosos e de rastreamento do câncer foram todos negativos. As funções renais melhoraram após reposição hídrica, pericardiocentese e descontinuação do tratamento com indometacina. Sua condição geral melhorou significativamente e nenhuma outra complicação foi observada. No 8º dia de internação, recebeu alta com enoxaparina subcutânea.

## Discussão

Este é o primeiro caso relatado de derrame pleuropericárdico maciço associado ao uso concomitante de dabigatrana e indometacina. Pelos motivos enumerados a seguir, pensamos que a toxicidade da dabigatrana teria sido a causa mais plausível de derrame pleuropericárdico no presente caso. (1) Presença de derrame pleuropericárdico hemorrágico, (2) desenvolvimento de derrames após desenvolvimento de insuficiência renal aguda, (3) altos níveis de APTT (91,4 segundos) e RNI (2,5) na internação^2^ e, finalmente, (4) ausência de outros motivos para explicar o derrame pleuropericárdico hemorrágico.

A dabigatrana é um metabólito ativo derivado da hidrólise do etexilato de dabigatrana. Inibe a trombina livre e ligada ao coágulo. A meia-vida da dabigatrana é de 12 a 14 horas, sendo excretada, em grande parte, pelos rins.^3^

As diretrizes atuais recomendam o acompanhamento regular da função renal nesses pacientes.^4^ No presente caso, o paciente apresentou insuficiência renal aguda após o início do tratamento com indometacina, um agente nefrotóxico, durante o tratamento com dabigatrana. Foram encontrados 12 casos de hemopericárdio associado à toxicidade da dabigatrana,^1 , 5 - 9^ A indicação para a dabigatrana foi a prevenção de acidente vascular cerebral na fibrilação atrial em todos os casos relatados. Em concordância com os nossos achados, 7 (58%) ADDIN EN.CITE^5 , 7 , 10 - 13^ desses casos apresentaram insuficiência renal aguda na internação e 4 (33%) ADDIN EN.CITE^5 , 7 - 9^ apresentaram hemopericárdio dois meses após o início da dabigatrana.

A absorção de etexilato de dabigatrana é mediada pela glicoproteína P (gpP). As interações da gpP no trato gastrointestinal podem interferir na absorção da dabigatrana. Ye CG et al. observaram que a indometacina pode inibir a gpP, diminuindo sua expressão e/ou a inibição direta de sua atividade.^14^ Assim, a coadministração com indometacina pode ter contribuído para a toxicidade da dabigatrana no nosso caso.

O idarucizumabe, um fragmento de anticorpo monoclonal humanizado que se liga à dabigatrana com alta afinidade sem aumentar os eventos trombóticos, é utilizado para reverter o efeito anticoagulante da dabigatrana em pacientes com condições hemorrágicas fatais.^15^ O efeito da dabigatrana pôde ser revertido com o uso do idarucizumabe no presente caso. Duas horas após a administração do idarucizumabe, verificou-se que o valor do APPT caiu de 91,4 segundos para 44 segundos. Além disso, não ocorreram complicações hemorrágicas ou trombóticas após a pericardiocentese.

## Conclusão

Deve-se considerar derrame pleuropericárdico em pacientes com dispneia desenvolvida recentemente, em tratamento com dabigatrana. O risco de sangramentos maiores pode aumentar quando a indometacina é utilizada concomitantemente com a dabigatrana. Ao se prescrever a dabigatrana, todos os pacientes devem ser informados sobre as possíveis interações com outros medicamentos. Os possíveis riscos de medicações nefrotóxicas utilizadas concomitantemente devem ser considerados em todos os pacientes em uso de dabigatrana e, se possível, esses agentes devem ser evitados, principalmente em pacientes com múltiplos fatores de risco de hemorragia. Por fim, os pacientes que desenvolvem hemorragia em uso de dabigatrana devem ser investigados quanto a co-medicações.
