# Supplementary material for: Uma Combinação Fatal: Indometacina e Dabigatrana
Source: Arq Bras Cardiol. 2020 May 11;114(4 Suppl 1):13–5. [Article in Portuguese] doi: 10.36660/abc.20180159 (PMC8149119; doi:10.36660/abc.20180159)
Supplement: Translation

# Translation: A Life-threatening Combination: Indomethacin and Dabigatran

**Authors:** Adem Adar, Orhan Onalan, Fahri Cakan

**Affiliations:** 1 Turkey Karabuk University Faculty of Medicine – Cardiology, Karabuk – Turkey

**Keywords:** Indomenthacin/administration & dosage, Dabigatran/administration & dosage, Cardiomegaly, Pleural Effusion, Renal Insufficiency, Echocardiography

## Introduction

Although rare, several bleeding complications may occur in patients receiving dabigatran. The risk of bleeding is particularly high in patients with impaired kidney functions or in patients who are on concomitant nephrotoxic drugs.^1^ We report a case of massive pleuropericardial effusion developed after the initiation of indomethacin treatment in a patient who was receiving dabigatran for deep venous thrombosis.

## Case Report

A 50-year old male patient admitted to the emergency department with progressive dyspnea. He had a heart rate of 120 beats/min, blood pressure of 180/90 mmHg, respiration rate of 15 breaths/min, oxygen saturation of 95% (on room air) and temperature of 36.8 ºС at presentation. He had a sedentary lifestyle, obesity (body mass index: 31 kg/m^2^), uncontrolled hypertension (for 5 years without medical therapy) and deep vein thrombosis (on dabigatran 150 mg twice a day for 50 days). Twenty days prior to his presentation, he started to receive indomethacin (once a day) for his leg pain. On physical examination, he had diminished heart and lung sounds. Electrocardiography showed sinus tachycardia. Cardiomegaly and bilateral pleural effusion (greater on the left lung) were noticed on chest X-ray. Chest computerized tomography confirmed bilateral pleural effusion and revealed massive pericardial effusion ( Figure 1A ). On admission, his blood tests were as follows: glucose: 107 mg/dL, urea: 63 mg/dL, creatinine: 1.99 mg/dL, AST: 69 U/L, ALT: 99 U/L, white blood cells: 9.73 10^9^/L, hemoglobin: 9.6 mg/dL, C-reactive protein: 0.9 mg/dL, activated partial thromboplastin time (APTT): 91.4-seconds and international normalized ratio (INR): 2.5. Since his last creatinine level was 1.1 mg/dL 20 days before (just before the initiation of indomethacin treatment), acute renal failure was considered. The patient was admitted to the intensive care unit and detailed echocardiography was performed. Transthoracic echocardiography revealed normal left ventricular systolic function (EF 65%), left ventricular concentric hypertrophy (LVMI: 118 g/m^2^), massive pericardial and pleural effusion ( Figure 1B ). There were no signs of cardiac tamponade on the first echocardiographic evaluation. However, during follow-up, his dyspnea and tachycardia were gradually increased, and right ventricular diastolic collapse was noticed on control echocardiography. We decided to perform urgent pericardiocentesis. In order to reduce the risk of bleeding, idarucizumab was administered (total 5 grams divided into two consecutive infusions of 2.5 grams) before pericardiocentesis. Two hours after administration of idarucizumab, the APTT value decreased to 44 seconds. Pericardiocentesis was performed with echocardiography guidance. Approximately 3L of blood-red, non-coagulating pericardial fluid was drained out ( Figure 2 ). Pericardial fluid analysis was negative for gram staining, cytology, polymerase chain reaction (PCR) and microorganisms ( *Mycobacterium tuberculosis* ). There was no pericardial effusion on repeat echocardiography performed on the following day after pericardiocentesis ( Figure 1C ). According to the modified Light criteria, pericardial effusion had exudative characteristics. Thoracentesis was then performed, and 2 L of pleural fluid was drained out. Biochemical tests were again consistent with exudative fluid. Inflammatory, rheumatological, infection and cancer screening markers were all negative. Renal functions improved after fluid replacement, pericardiocentesis, and discontinuation of indomethacin therapy. His overall status improved significantly, and no other complications were noticed. On the 8^th^day of hospitalization, he was discharged with subcutaneous enoxaparin.

**Figure 1:** – A) Massive pericardial and pleural effusion in thoracic tomography. B) Pericardial effusion surrounding the heart and pleural effusion. C) Pericardial effusion was drained completely.

**Figure 2:** – Blood-red non-coagulating pericardial fluid.

## Discussion

This is the first reported case of massive pleuropericardial effusion associated with concomitant use of dabigatran and indomethacin. For the following reasons, we thought that dabigatran toxicity was the most plausible cause of pleuropericardial effusion in the present case. (1) Presence of hemorrhagic pleuropericardial effusion, (2) development of effusions after development of acute renal failure, (3) high APTT (91.4 seconds) and INR (2.5) levels at presentation,^2^ and, finally, (4) no other reasons to explain hemorrhagic pleuropericardial effusion.

Dabigatran is an active metabolite derived from the hydrolysis of dabigatran etexilate. It inhibits both free and clot-bound thrombin. The half-life of dabigatran is 12–14 hours and it is largely excreted via the kidneys.^3^

Current guidelines recommend regular follow-up of kidney function in these patients.^4^ In the present case, the patient experienced acute renal failure after initiation of indomethacin, a nephrotoxic agent, during dabigatran therapy. We found 12 cases of hemopericardium associated with dabigatran toxicity.^1 , 5 - 9^ Indication for dabigatran was stroke prevention in atrial fibrillation for all reported cases. Consistent with our findings, 7 (58%)^, 7 , 10 - 13^ of these cases had acute renal failure at presentation and 4 (33%)^5 , 7 - 9^ experienced hemopericardium two months after initiation of dabigatran.

The absorption of dabigatran etexilate is mediated by the p-glycoprotein (P-gp). Gastrointestinal tract-based P-gb interactions may interfere with the absorption of dabigatran. Ye CG et al. reported that indomethacin may inhibit P-gp by decreasing its expression and/or direct inhibition of its activity.^14^ Thus, co-administration with indomethacin may have contributed to dabigatran toxicity in our case.

Idarucizumab, a humanized monoclonal antibody fragment which binds to dabigatran with high affinity without increasing thrombotic events, is used for reversing the anticoagulant effect of dabigatran in patients with life-threatening bleeding conditions.^15^ The effect of dabigatran was successfully reversed with idarucizumab in the present case. Two hours after initiating idarucizumab, the APPT value was found to fall from 91.4 seconds to 44 seconds. In addition, no bleeding or thrombotic complications occurred after pericardiocentesis.

## Conclusion

Pleuropericardial effusion should be considered in patients with newly developed dyspnea who are under dabigatran treatment. The risk of major bleeding may increase when indomethacin is used concomitantly with dabigatran. When prescribing dabigatran, all patients should be informed about the potential interactions with other drugs. Potential risks of concomitant nephrotoxic medications should be considered in all patients receiving dabigatran and, if possible, these agents should be avoided, particularly in patients with multiple risk factors for bleeding. Finally, patients who develop bleeding under treatment with dabigatran should be investigated for co-medications.
